# Consensus Kernel *K*-Means Clustering for Incomplete Multiview Data

**DOI:** 10.1155/2017/3961718

**Published:** 2017-10-22

**Authors:** Yongkai Ye, Xinwang Liu, Qiang Liu, Jianping Yin

**Affiliations:** ^1^College of Computer, National University of Defense Technology, Changsha, China; ^2^State Key Laboratory of High Performance Computing, National University of Defense Technology, Changsha, China

## Abstract

Multiview clustering aims to improve clustering performance through optimal integration of information from multiple views. Though demonstrating promising performance in various applications, existing multiview clustering algorithms cannot effectively handle the view's incompleteness. Recently, one pioneering work was proposed that handled this issue by integrating multiview clustering and imputation into a unified learning framework. While its framework is elegant, we observe that it overlooks the consistency between views, which leads to a reduction in the clustering performance. In order to address this issue, we propose a new unified learning method for incomplete multiview clustering, which simultaneously imputes the incomplete views and learns a consistent clustering result with explicit modeling of between-view consistency. More specifically, the similarity between each view's clustering result and the consistent clustering result is measured. The consistency between views is then modeled using the sum of these similarities. Incomplete views are imputed to achieve an optimal clustering result in each view, while maintaining between-view consistency. Extensive comparisons with state-of-the-art methods on both synthetic and real-world incomplete multiview datasets validate the superiority of the proposed method.

## 1. Introduction

The term “multiview data” refers to data that have different sources or modalities. Each source or modality is considered as one “view,” and different views have different physical meanings and statistical properties. For example, a web page can be described by the pictures and text it contains, while a news story may be reported by different sites each with its own different viewpoints. A significant number of studies aimed to investigate and learn from multiple views in the past [1, 2]. Multiview clustering, which is one component of multiview learning, aims at grouping samples by utilizing information from different views. Extensive research has been conducted into multiview clustering; these can be roughly categorized into early fusion approaches and late fusion approaches. Early fusion approaches fuse the multiview information in an early stage of the process and then perform clustering [3–9], while late fusion approaches group data by fusing previously clustered results from separate views [10, 11].

However, in real-world applications, some views may be incomplete for a variety of reasons, which hurts the clustering performance of multiview data. For example, in the context of patient grouping, the data from different tests can serve as different views. If a test is too expensive, some patients may be unable to afford it, which leads to an incomplete view for this particular test. Similarly, in webpage clustering, image data and text data are two modalities that represent a page; however, some pages may not contain any images, which makes the data for the image view incomplete.

Existing studies of incomplete multiview clustering can be roughly divided into two categories: subspace methods and imputation methods. The method outlined in [12], which was the first subspace method for incomplete multiview clustering, learns the common subspace of two views via nonnegative matrix factorization. Several variants of this method were proposed following its introduction. In [13], feature learning is integrated into the subspace learning process and the assumption that the data is nonnegative is not required. The method proposed in [14] learns a latent global graph representation and the subspace simultaneously by adding a novel Laplacian graph regularization term. The other important category of method for incomplete multiview clustering is imputation methods, which handle incomplete views by filling in the missing parts. The method proposed in [15] fills the kernel of an incomplete view according to the Laplacian regularization of the other complete view. Subsequently, the method proposed in [16] tackles the situation where two views are incomplete by alternately updating one view according to the other view. In [17], the incomplete views are imputed via low rank decomposition. As different views are assumed to be generated from a shared subspace, the data matrices of different views can be decomposed using a common factor. Most of these imputation methods simply execute a conventional multiview clustering algorithm after filling the incomplete views. Most recently, a method was proposed in [18], whereby the imputation is not separated from the multiview clustering process. More specifically, the imputation and the multiple kernel clustering are integrated into a unified procedure for better clustering performance.

Integrating imputation and multiview clustering into a unified learning process makes the imputation better serve the clustering objective. This advantage helps the method in [18] to outperform other methods that perform imputation and clustering separately. However, the disadvantage of the method in [18] is that multiview clustering solution it proposes overlooks the consistency between views, which may reduce the final clustering performance. In [18], multiview clustering is achieved by learning a linear combination of kernels that reaches the optimal kernel *k*-means clustering result. Consequently, the linear combination to build the best kernel for clustering is learned without considering the relationships between views. Similarly, the imputation is guided only by the clustering objective and the consistency between views is neglected. However, the consistency between views is one of the inherent properties of multiview data [1]; if this critical property is not considered, the learning of the linear combination of kernels and the imputation in [18] may lead to poor clustering performance. Previous research into multiview clustering has shown that considering the consistency between views helps to boost the performance of multiview clustering [3]. In this study, we wish to build on the advances made in [18] while also considering the consistency between views in order to further improve clustering performance. Therefore, we propose a novel incomplete multiview clustering method that simultaneously fills the incomplete kernels from incomplete views and learns a consistent clustering result. To model the between-view consistency, the similarity between the consistent clustering result and the clustering result of each view is calculated. The consistency between views is measured by the sum of these similarities. The missing parts of kernels and the consistent clustering result are learned in order to achieve the optimal clustering result in each view while keeping consistency between views. Here, the learning process considers both the data structures within views and the consistent relations between views, which benefits the multiview clustering performance. The proposed objective function is then solved by alternately optimizing partial variables. Each subproblem that optimizes the corresponding partial variables either can be solved by means of eigenvector decomposition or has a closed-form solution. To evaluate the performance of the proposed method, we compare it with state-of-the-art methods on three synthetic and one real-world incomplete multiview datasets. Empirical results validate the superiority of the proposed method for incomplete multiview clustering.

The main contributions of this paper can be summarized as follows:

1. We propose a novel incomplete multiview clustering method, which simultaneously learns a consistent clustering decision and fills the incomplete kernels from incomplete views with explicit modeling of between-view consistency.
2. We design an alternating optimization algorithm to solve proposed method's optimization problem. Here, the optimization problem is divided into three subproblems. The subproblems either can be solved by means of eigenvector decomposition or has a closed-form solution.
3. We also provide thorough convergence analysis of the alternating optimization algorithm, including theoretical proof and empirical validations.

## 2. The Proposed Method

Regarding the consistency, we propose that a consistent clustering decision be learned that is similar to each view's kernel *k*-means clustering result. To handle the incomplete views, we simultaneously fill the incomplete views and learn the consistent clustering decision. In the following subsections, we first introduce the notation used in problem formulation, after which kernel *k*-means is briefly reviewed. We then outline how a consistent decision might be found. Next, we introduce the objective function of our method to explain how the kernel filling and decision learning processes are integrated. Finally, we analyse the convergence of the proposed algorithm.

### 2.1. Notation

Assume that there are *N* samples and *P* views for the multiview data. For clarity, sample's information in a view is referred to as an instance of the sample in this paper. For incomplete multiview data, a sample's instance in a view could be missing. **S** is an *N* × *P* zero-one matrix that indicates which instances are missing; when **S**_*ij*_ = 0, sample *i*'s instance in view *j* is missing. **S**_*j*_ denotes the *j*th column of **S**. Because our method is based on kernel *k*-means, we assume that the input multiview data is kernel data. For each view *j*, we have a *N* × *N* kernel matrix **K**_*j*_. The details of how kernel data are built can be found in Section 3.1, where datasets used in this paper are introduced.

**Figure 1:**
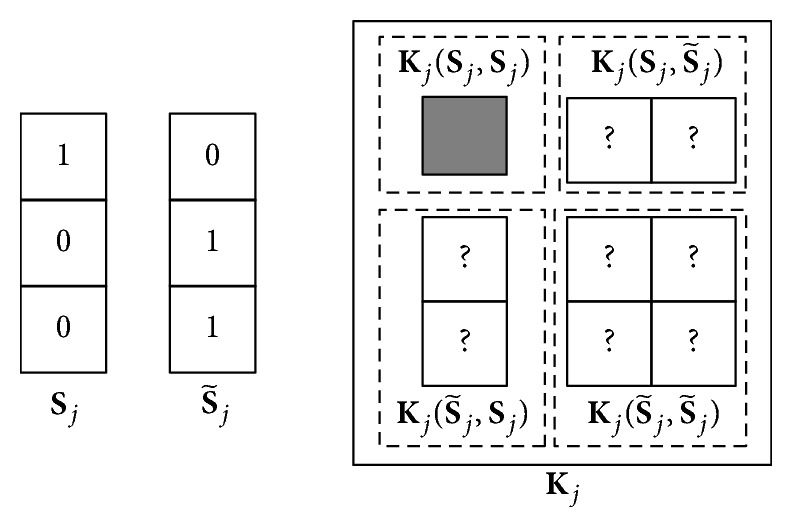
A simple example of notation. **S**_*j*_ indicates that only the instance of the first sample is visible in view *j*. ${\tilde{S}}_{j}$ is derived from **S**_*j*_. The kernel matrix of view *j* is **K**_*j*_. **K**_*j*_ can be divided into four parts according to **S**_*j*_ and ${\tilde{S}}_{j}$. It is notable that only **K**_*j*_(**S**_*j*_, **S**_*j*_) is visible.

In a view *j*, some instances may be missing, which will lead to an incomplete kernel **K**_*j*_. To describe the visible and missing parts of the incomplete kernel **K**_*j*_, we define an operator **K**(row*S*, col*S*), which selects corresponding rows and columns of **K** according to zero-one vectors row*S* and col*S* (where 1 indicates selected). Moreover, we define ${\tilde{S}}_{j}=1-S_{j}$. Thus **K**_*j*_(**S**_*j*_, **S**_*j*_) is the visible part of the kernel matrix, while $K_{j}(S_{j},{\tilde{S}}_{j})$, $K_{j}({\tilde{S}}_{j},S_{j})$, and $K_{j}({\tilde{S}}_{j},{\tilde{S}}_{j})$ are the missing parts.  Figure 1 shows a simple example of notation with three samples.

### 2.2. Kernel *k*-Means

Here, kernel *k*-means refers to the *k*-means method developed for kernel data. Define a mapping from *𝒳* to a reproducing kernel Hilbert space *ℋ* : *ϕ*(·) : *x* ∈ *𝒳* → *ℋ*. {*x*_*i*_}_*i*=1_^*n*^ ∈ *𝒳* is the sample set. A zero-one matrix **Z** ∈ {0,1}^*N*×*K*^ is used to store cluster information, where **Z**_*ic*_ = 1 indicates that sample *i* is in cluster *c*. The *k*-means objective in kernel space is as follows:

(1) minZ∈0,1N×K ∑c=1K∑i=1NZicϕxi−μc22s.t. ∑c=1KZic=1,i=1,…,N,

where *μ*_*c*_ is the average of samples in cluster *c*. The number of samples in cluster *c* is *N*_*c*_ = ∑_*i*=1_^*N*^**Z**_*ic*_, such that *μ*_*c*_ = (1/*N*_*c*_)∑_*i*=1_^*N*^**Z**_*ic*_*ϕ*(*x*_*i*_). The kernel matrix is denoted by **K**, where **K**_*ij*_ = *ϕ*(*x*_*i*_)^*T*^*ϕ*(*x*_*j*_). Define matrix **L** = diag⁡([*N*_1_^−1^, *N*_2_^−1^,…, *N*_*k*_^−1^]), so that the equivalent matrix form of (1) is as follows:

(2) minZ∈0,1N×K trK−trL1/2ZTKZL1/2s.t. Z1K=1N,

where tr(·) is the trace operator and 1_*K*_ is a *K*-length vector in which all elements are 1.

The discreteness of **Z** makes (2) difficult to solve. An approximated problem that is easier to solve can be arrived at by relaxing the discreteness constraints on **Z**. By denoting **U** = **ZL**^1/2^, the approximated problem can be expressed as follows:

(3) minU∈RN×K trKI−UUTs.t. UTU=I.

The optimal **U** can be solved by obtaining eigenvectors corresponding to *k* larger eigenvalues of **K** [9]. Although **U** contains the cluster indicator information, *k*-means should be performed on **U** to recover the actual clustering label.

### 2.3. Finding the Consistent Decision

So as to consider the consistency between views, we propose to find a consistent clustering decision according to the clustering results of different views. Suppose **U**_*j*_ is the eigenvector matrix found by kernel *k*-means in view *j*. **U**_*j*_, while not the actual clustering label of view *j*, does store the cluster information. Accordingly, we can find a matrix **U**^*∗*^ that is consistent with all **U**_*j*_ and then recover the final decision from **U**^*∗*^.

To find the consistent **U**^*∗*^, it is necessary to define the similarity between **U**^*∗*^ and **U**_*j*_. Inspired by [3, 19], the similarity is defined as

(4) $$\begin{array}{c} L\left(\;U_{j},U^{*}\;\right)=\langle \;\frac{U_{j}U_{j}^{T}}{{\left\|U_{j}U_{j}^{T}\right\|}_{F}},\frac{U^{*}{U^{*}}^{T}}{{\left\|U^{*}{U^{*}}^{T}\right\|}_{F}}\;\rangle , \end{array}$$

where ‖·‖_*F*_ is the Frobenius norm. Adding regularization **U**^*∗*^^*T*^**U**^*∗*^ = **I** on **U**^*∗*^, we have

(5) $$\begin{array}{c} L\left(\;U_{j},U^{*}\;\right)=tr\left(\;U_{j}U_{j}^{T}U^{*}{U^{*}}^{T}\;\right). \end{array}$$

There may be other possible definitions of similarity between **U**_*j*_ and **U**^*∗*^. However, (4) is chosen because it allows an easy alternating optimization for the proposed method.

As expected that the consistent decision should be similar to the kernel *k*-means result of each view, we maximize the sum of similarities to find the consistent decision as follows:

(6) $$\begin{array}{c} \overset{P}{\underset{j=1}{\sum }}L\left(\;U_{j},U^{*}\;\right)=\overset{P}{\underset{j=1}{\sum }}tr\left(\;U_{j}U_{j}^{T}U^{*}{U^{*}}^{T}\;\right). \end{array}$$

It is notable that each view is considered to be equally important in (6). If the importance of each view is prior knowledge, we can weigh the views differently and adapt (6) to a weighted sum of similarities. However, in this paper, we maintain the same weight for all views for model simplicity. Although learning the accurate weights of views is valuable under circumstances where there are some views with heavy noise, it is beyond the scope of this paper.

### 2.4. Objective Function

If all views are complete, it is easy to find the consistent decision by maximizing (6). When some views are incomplete, however we need to fill the corresponding kernels of those views for kernel *k*-means. We expect that these filled kernels will lead to better clustering in each view and a consistent decision. In other words, the kernel filling is guided by both the clustering objective in each view and the consistency between views. So the filling procedure considers both the data structure within each view and the relationship between views. To achieve this, we propose the objective function as follows:

(7) minUj,U∗,Kj ∑j=1PtrKjI−UjUjT−β∑j=1PLUj,U∗s.t. UjTUj=I,∀j=1,…,P, U∗TU∗=I, KjSj,Sj=K^jSj,Sj,∀j=1,…,P, Kj⪰0,∀j=1,…,P,

where *L*(**U**_*j*_, **U**^*∗*^) = tr(**U**_*j*_**U**_*j*_^*T*^**U**^*∗*^**U**^*∗*^^*T*^). **K**_*j*_ is the kernel that needs to be learned, which should be positive semidefinite. ${\hat{K}}_{j}(S_{j},S_{j})$ is the visible part of the original kernel data. The third constraint actually forces **K**_*j*_(**S**_*j*_, **S**_*j*_) to be the same as the original kernel data. However, $K_{j}({\tilde{S}}_{j},S_{j})$, $K_{j}(S_{j},{\tilde{S}}_{j})$, and $K_{j}({\tilde{S}}_{j},{\tilde{S}}_{j})$ still need to be optimized.

It is notable that the objective function consists of two parts. ∑_*j*=1_^*P*^tr[**K**_*j*_(**I** − **U**_*j*_**U**_*j*_^*T*^)] is the sum of kernel *k*-means objective in each view, and ∑_*j*=1_^*P*^*L*(**U**_*j*_, **U**^*∗*^) is the term designed to model between-view consistency. A parameter *β* is added to balance the importance of single view clustering performance and the consistency between views.

**Remark 1 .**

Like the method proposed in [18], our method simultaneously fills incomplete kernels and performs multiview clustering. However, there are also major differences between the two methods. In [18], multiview clustering is achieved by learning the best combination of kernels for the best clustering performance, which overlooks the consistency between views. Differently, our method learns a consensus clustering decision from each view's kernel *k*-means result, which explicitly models the consistency. More importantly, our method does not simply revise the method in [18] incrementally by adding a consistency regularization term; instead, we propose a new objective function that inherits the advantages of simultaneously performing imputation and multiview clustering.

**Remark 2 .**

The strategy of learning consistent clustering decision was also applied in [3, 19]. The former work is based on spectral clustering, while the latter one is based on kernel *k*-means. But it is worth noting that these works cannot deal with the incomplete multiview situation.

### 2.5. Optimization

Optimizing all variables of (7) in one step is difficult. Instead, we develop an algorithm to solve the problem where **U**_*j*_, **U**^*∗*^, and **K**_*j*_ are optimized alternatively. The optimal solutions of the subproblems can be found easily, and the whole alternating updating process is guaranteed to converge to a local minimum.

#### 2.5.1. Updating **U**_*j*_

When we only optimize **U**_*j*_, the subproblem has a similar form to kernel *k*-means and can be solved by means of eigenvalue decomposition in a similar way. The subproblem of updating **U**_*j*_ is as follows:

(8) maxUj trUjTKj+βU∗U∗TUjs.t. UjTUj=I.

#### 2.5.2. Updating **U**^*∗*^

Similarly, the subproblem of updating **U**^*∗*^ can be solved by means of eigenvalue decomposition after reformulation. The subproblem of updating **U**^*∗*^ is as follows:

(9) maxU∗ ∑j=1PtrUjUjTU∗U∗Ts.t. U∗TU∗=I.

Equation (9) is equivalent to the following optimization problem:

(10) maxU∗ trU∗T∑j=1PUjUjTU∗s.t. U∗TU∗=I.

#### 2.5.3. Updating **K**_*j*_

The subproblem for **K**_*j*_ is an optimization problem with positive semidefinite constraint. Let **V**_*j*_ = **I** − **U**_*j*_**U**_*j*_^*T*^, so that the subproblem is as follows:

(11) minKj trKjVjs.t. KjSj,Sj=K^jSj,Sj, Kj⪰0.

Because **K**_*j*_ is positive semidefinite, **K**_*j*_ can be decomposed as **A**_*j*_**A**_*j*_^*T*^, where **A**_*j*_ is a *N* × 1 vector [15, 18]. If we obtain **A**_*j*_, **K**_*j*_ can be recovered.

For clarity, we divide **A**_*j*_ into two parts: **A**_*j*_^*v*^ = **A**_*j*_(**S**_*j*_, 1) and $A_{j}^{m}=A_{j}({\tilde{S}}_{j},1)$. **A**_*j*_^*v*^ is selected according to the indexes of the visible instance in view *j*, and **A**_*j*_^*m*^ is selected according to the indexes of the missing instance in view *j*. Therefore, the kernel matrix of view *j* can be divided into four parts as follows:

(12) Kjvv=KjSj,Sj=AjvAjvT,Kjvm=KjSj,S~j=AjvAjmT,Kjmv=KjS~j,Sj=AjmAjvT,Kjmm=KjS~j,S~j=AjmAjmT.

It is notable that **K**_*j*_^*vv*^ is the only visible part. The *N* × *N* matrix **V**_*j*_ can be divided into four corresponding parts in a similar way to **K**_*j*_. According to the first constraint in (11), $A_{j}^{v}{A_{j}^{v}}^{T}={\hat{K}}_{j}(S_{j},S_{j})$. To obtain **A**_*j*_^*m*^, we have a problem equivalent to (11) as follows:

(13) minAjm trAjv;AjmTVjvvVjvmVjmvVjmmAjv;Ajm.

Taking the derivative of (13), we can obtain the closed-form solution for **A**_*j*_^*m*^:

(14) $$\begin{array}{c} A_{j}^{m}=-{\left(\;V_{j}^{vm}{V_{j}^{mm}}^{-1}\;\right)}^{T}A_{j}^{v}. \end{array}$$

By denoting **V**_*j*_^*vm*^**V**_*j*_^*mm*^^−1^ as **V**_*j*_^*vm*/*mm*^, the missing parts of **K**_*j*_ can be calculated as

(15) Kjvm=−K^jSj,SjVjvm/mm,Kjmv=−Vjvm/mmTK^jSj,Sj,Kjmm=Vjvm/mmTK^jSj,SjVjvm/mm.

The overall optimization process is summarized in Algorithm 1.

### 2.6. Convergence Property

In this subsection, we provide a theoretical proof of the convergence of the proposed optimization algorithm. First, we need to prove that the objective value of (7) is lower-bounded.

**Lemma 3 .**

if **K**⪰0, **U**^*T*^**U** = **I**, then tr[**K**(**I** − **UU**^*T*^)] ≥ 0.

**Proof**

Denoting **U** = [**u**_1_,…, **u**_*K*_], because **U**^*T*^**U** = **I**, we have **UU**^*T*^**U** = **U**. So **UU**^*T*^**u**_*j*_ = **u**_*j*_, 1 ≤ *j* ≤ *K*. This implies that **UU**^*T*^ has *K* eigenvalues with 1. Moreover, because the rank of **UU**^*T*^ is not larger than *K*, the remaining *N* − *K* eigenvalues are 0.

Therefore, **I** − **UU**^*T*^ is positive semidefinite and can thus be decomposed as **vv**^*T*^. Because **K**⪰0, tr[**K**(**I** − **UU**^*T*^)] = tr(**Kvv**^*T*^) = **v**^*T*^**Kv** ≥ 0.

**Lemma 4 .**

One has the following:

(16) $$\begin{array}{c} -tr\left(\;U_{j}U_{j}^{T}U^{*}{U^{*}}^{T}\;\right)\geq -N. \end{array}$$

**Proof**

According to the definitions of Frobenius norm and trace, we have the following:

(17) UjUjT−U∗U∗TF2=trUjUjT−U∗U∗T2=trUjUjT2+trU∗U∗T2−2trUjUjTU∗U∗T.

Following the constraints in (7), we have **U**_*j*_^*T*^**U**_*j*_ = **I** and **U**^*∗*^^*T*^**U**^*∗*^ = **I**. So, tr[(**U**_*j*_**U**_*j*_^*T*^)^2^] = tr[(**U**^*∗*^**U**^*∗*^^*T*^)^2^] = tr(**I**) = *N*. Finally, we have −tr(**U**_*j*_**U**_*j*_^*T*^**U**^*∗*^**U**^*∗*^^*T*^) = (1/2)‖**U**_*j*_**U**_*j*_^*T*^ − **U**^*∗*^**U**^*∗*^^*T*^‖_*F*_^2^ − *N* ≥ −*N*.

According to Lemmas 3 and 4, the objective value of (7) is lower-bounded. Moreover, because we obtain the optimal solution to the corresponding subproblem in each step of the alternate updating, the objective value of (7) is therefore nonincreasing during this process. Since the objective value is lower-bounded and nonincreasing, the alternate updating algorithm is guaranteed to converge.

## 3. Experiments

### 3.1. Datasets

One incomplete multiview dataset and three complete multiview datasets are used in the experiments, as shown in Table 1. 3 Sources, the incomplete multiview dataset, has been compiled from three news sites: BBC, Reuters, and the Guardian. The dataset contains 416 news stories, and articles for some stories are missing from each site. More information about 3 Sources can be found in Table 2. Artificial incomplete multiview data are generated from complete multiview datasets using a random missing mechanism. The details of the generating process can be found in Section 3.3. For Digital (https://github.com/HoiYe/DigitalDataset), Flower 17 (http://www.robots.ox.ac.uk/~vgg/data/flowers/17/) and Flower 102 (http://www.robots.ox.ac.uk/~vgg/data/flowers/102/), and precomputed kernel matrices are used. As for 3 Sources, we generate Gaussian kernels with widths set as the mean of sample pair distances.

### 3.2. Compared Methods

The proposed method is compared with three state-of-the-art methods including one of the latest imputation methods and two representative subspace methods. The best clustering result of a single view and the multiview clustering result with zero-filling kernels, as important baselines, are also compared.

*Best Result of a Single View (BSV)*. We perform clustering with the remaining samples in each view and choose the best. Because the view is incomplete, the missing samples are assigned random labels, after which the overall performance is reported.

*Multiple Kernel k-Means (MKKM)*. Multiple kernel *k*-means is applied to the zero-filling kernels.

*Multiple Kernel k-Means with Incomplete Kernels (MKKIK)*. The algorithm proposed in [18] learns the missing parts and performs multiple kernel *k*-means simultaneously.

*Partial View Clustering (PVC)*. The subspace method proposed in [12], which learns a subspace where two views' instances of the same sample are similar.

*Incomplete Multimodal Visual Data Grouping (IMG)*. The subspace method proposed in [14], which added a graph Laplacian term to learn a latent global graph representation and the subspace simultaneously.

*k-Means-Based Consensus Clustering (KCC)*. The work in [20] proposed a unified framework for *k*-means-based consensus clustering that can handle cases with incomplete partitions. Although this work does not focus on incomplete multiview clustering specifically, if we use the clustering results of each of the views as input partitions, it can deal with incomplete multiview clustering.

### 3.3. Experimental Settings

In our experiments, the number of clusters is set as the true number of classes. Kernels are centralized and scaled during the preprocessing procedure following the suggestion put forward in [21]. Incomplete multiview data is manually produced for the complete multiview datasets. If the incomplete samples ratio (ISR) is *ϵ*, then *ϵ* × *N* samples are randomly selected as incomplete. We keep the probability that a view is missing set at *q*_0_ = 0.5. A random vector **g** = (**g**_1_,…, **g**_*P*_) ∈ [0,1]^*P*^ is generated for each incomplete sample. The *p*th view of an incomplete sample exists only if **g**_*p*_ > *q*_0_. Because at least one view should always exist for a sample, a random vector is accepted until there is one view available for this sample. *ϵ* is varied from 0.1 to 0.9 to produce different missing patterns. For each value of *ϵ*, 10 random missing patterns are generated and the average performance reported. For the proposed method, the parameter *β* is searched for in [10^−5^, 10^−4^,…, 10^4^, 10^5^]/*P*. *P* represents the number of views, which is divided to avoid the scale difference caused by view number. For the relatively large dataset Flower 102, we search a smaller range: [10^−3^, 10^−2^,…, 10^2^, 10^3^]/*P*. For PVC, we use the code provided by the authors, and the parameter is tuned from [10^−6^, 10^5^,…, 1] following the suggestion in [12]. For IMG, the same parameter as in PVC is set as the tuned value in PVC, and the other two parameters are set as advised in [14]. We use normalized mutual information (NMI) as the clustering evaluation [3, 12].

### 3.4. Experimental Results

**Figure 2:**
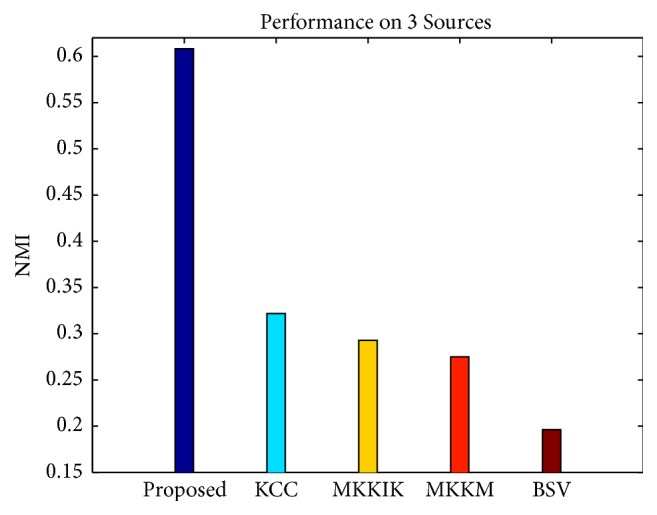
Performance comparison on real-world dataset in terms of NMI.

Figure 2 shows the results on 3 Sources, the real-world incomplete multiview dataset. BSV performs worse as it only considers information from one view. Using the multiview information fusion, MKKM with zero-filling reaches a better NMI than BSV, while MKKIK outperforms MKKM for a more reasonable imputation. The proposed method achieves a significant NMI boost of about 30% compared with MKKIK. Our method fills the incomplete kernels to make the clustering result of each view consistent, while MKKIK does not consider the consistency. We suggest that there may be a strong underlying consistency between views on 3 Sources, so the proposed method outperforms MKKIK in part due to the fact that this consistency is considered. Moreover, our method also outperforms KCC, which is a method that does consider the consistency; we suggest that this occurs because KCC does not have an imputation process. In KCC, the consensus clustering decision is learned from the remaining incomplete partitions.

**Figure 3:**
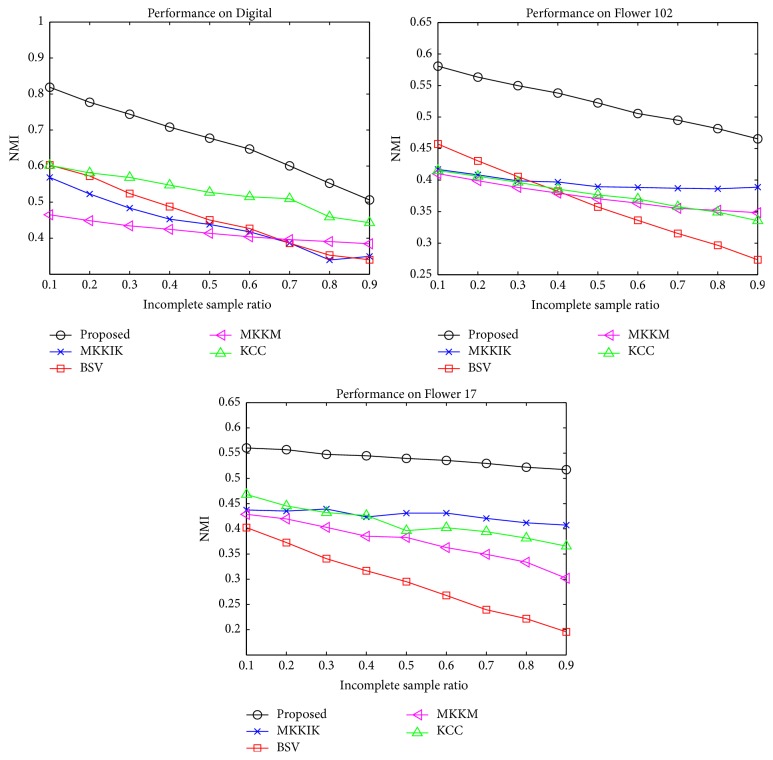
Performance comparison on synthetic incomplete multiview datasets in terms of NMI.

**Figure 4:**
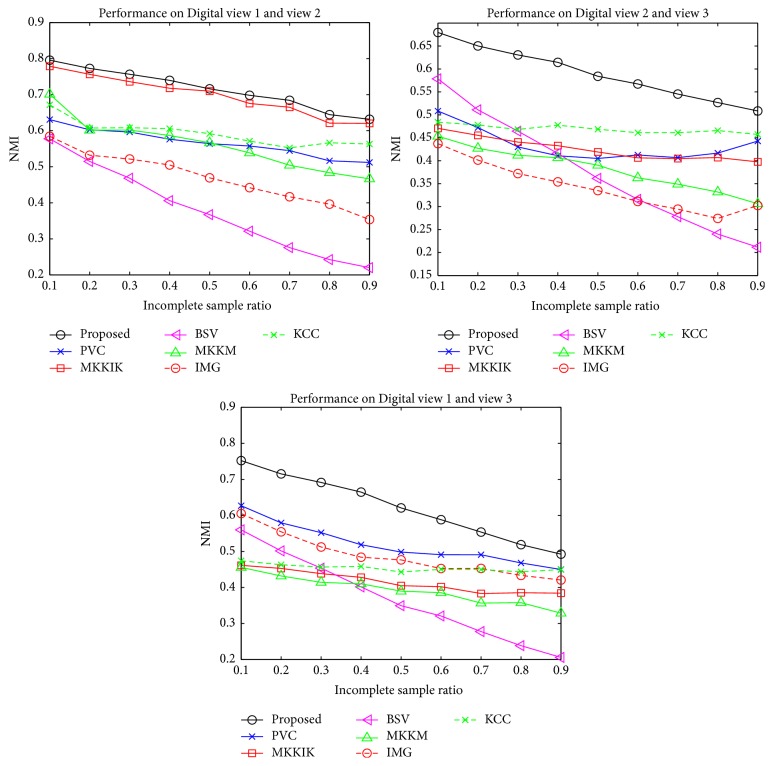
Performance comparisons on synthetic incomplete two-view data from Digital in terms of NMI.

Figure 3 summarizes the results on the three artificial incomplete multiview datasets: Flower 17, Flower 102, and Digital. It can be observed that the proposed method constantly achieves the best NMI compared with the state-of-the-art methods with different ISRs. Moreover, the proposed method significantly outperforms the second-best method with different ISRs. For example, the proposed method outperforms the second-best method by around 20% on Digital when ISR is 0.1. It is also notable that when ISR increases, the performance of all methods decreases, which validates the degenerating effect of incomplete views. In Figure 4, we compare our method with two additional methods, PVC and IMG, which are representative subspace methods that focus on two views. We report the results on the view pairs of Digital. The proposed method constantly exhibits better performance than all other methods on all view pairs. This shows that the proposed method can also perform better than the state-of-the-art subspace methods in a two-view situation.

To summarize, the proposed method demonstrates its superiority against the state-of-the-art methods on both synthetic and real-world multiview datasets. We suggest that the imputation in the proposed method considers both clustering performances in each view and the consistency between views, which contributes to the superiority of the proposed method.

### 3.5. Convergence Study

**Figure 5:**
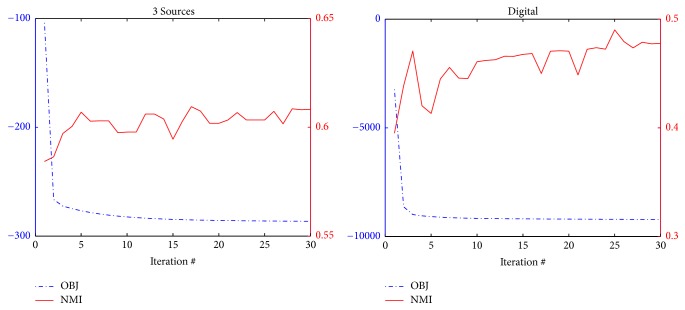
Objective value curve and NMI curve against iteration number.

As was proved in the previous section, the proposed algorithm is guaranteed to be convergent. Here we empirically validate the convergence property, as illustrated in Figure 5. Due to space limitations, we show the objective curve and NMI curve when incomplete sample ratio is 0.9 for 3 Sources and Digital. The objective values decrease as the iteration number increases, and the objective values converge within 30 iterations. Although the NMIs do not grow monotonically, they achieve relatively large value when the number of iterations reaches 30.

### 3.6. Parameter Study

**Figure 6:**
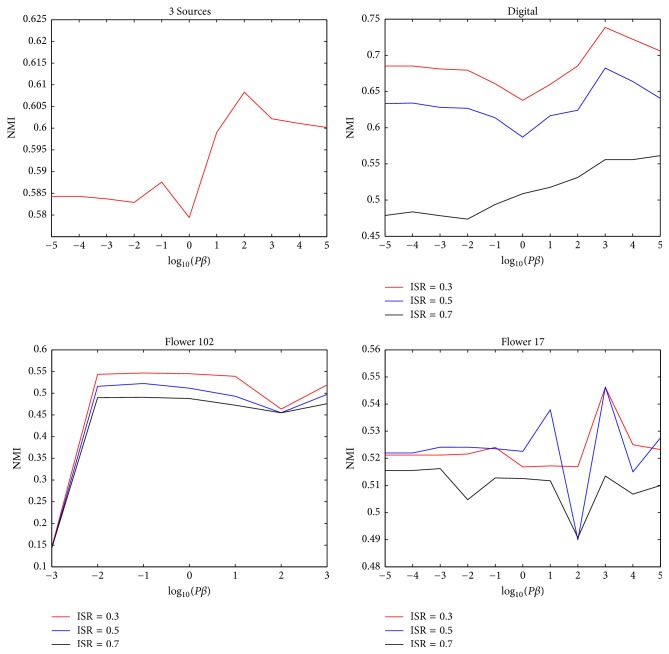
Parameter studies on different datasets.

Figure 6 illustrates how parameter *β* influences clustering performance. On Digital, Flower 102, and Flower 17, we present the performance curves for three ISRs: 0.3, 0.5, and 0.7. On 3 Sources, performance is optimal when *β* = 10^2^/*P*. On Digital, the performance remains relatively stable as the parameter changes. On Flower 102, the performance maintains a relatively high level when *β* is greater than 10^−2^/*P*. On Flower 17, the performance is sensitive to the parameter when *β* is larger than 10/*P*. Overall, across the four datasets, the performance tends to be better when *β* is larger. According to (7), when *β* is larger, the clustering results between views should have greater consistency. Thus, better performance when *β* is larger indicates relatively strong consistency between views on these datasets. It should also be emphasized that although the performance on Flower 17 is relatively sensitive to *β*, Figure 3 indicates that the proposed method still outperforms other methods for worse choices of *β*. When applying the proposed method on other datasets, we recommend a comparatively large value of *β* in cases when views share a substantial amount of common information.

## 4. Conclusion

In this paper, we have proposed a consensus kernel *k*-means clustering method for incomplete multiview data in which a consensus clustering decision and the missing parts of the incomplete kernels are learned. In this way, the imputation of incomplete kernels leads to better clustering of each view and maintains consistency between views, which benefits the final clustering decision. Comprehensive experiments validate the clustering performance improvement of the proposed method compared with state-of-the-art methods.

## Figures and Tables

**Algorithm 1:**
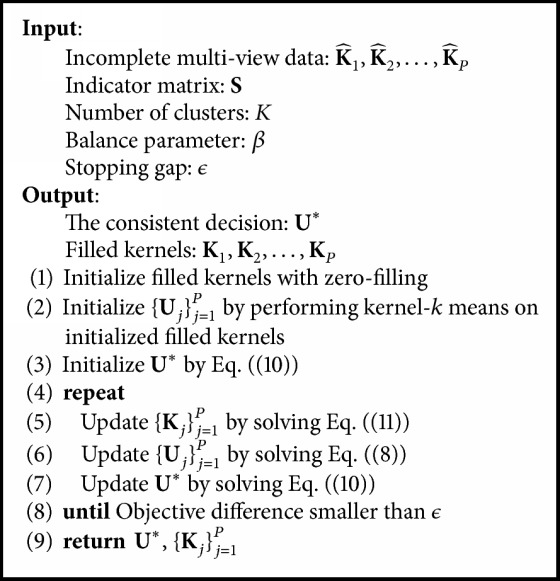
Consensus kernel *k*-means clustering for incomplete multiview clustering.

**Table 1:** Overview of datasets.

Dataset	Number of samples	Number of views	Number of clusters
3 Sources	416	3	6
Digital	2000	3	10
Flower 17	1360	7	17
Flower 102	8189	4	102

**Table 2:** Details of the 3 Sources dataset.

	Articles	Missing ratio
BBC	352	0.1538
The Guardian	302	0.2740
Reuters	294	0.2933
